# “Old” and “New” Media Discourses on Chinese Outbound Tourism to Switzerland Before and During the Covid-19 Outbreak. An Exploratory Study

**DOI:** 10.1007/978-3-030-65785-7_50

**Published:** 2020-11-28

**Authors:** Lea Hasenzahl, Lorenzo Cantoni

**Affiliations:** 1 Department for Informatics, Technical University of Munich, Garching bei München, Bayern Germany; 2 Smart Tourism Education Platform (STEP) College of Hotel and Tourism Management, Kyung Hee University, Seoul, Korea (Republic of); 3 Department of Tourism and Service Management, MODUL University Vienna, Vienna, Wien Austria; Institute of Digital Technologies for Communication (ITDxC), Università della Svizzera italiana, Lugano, Switzerland

**Keywords:** Thematic analysis, Media analysis, Chinese outbound tourism, Visitor-host-relationship, Sustainable tourism, Tourism discourses

## Abstract

The paper presents an exploratory research focused on the themes concerning Chinese Outbound Tourism to Switzerland in the period from January 2019 to June 2020 including the Covid-19 outbreak. It analyses news media articles from Swiss-German print media covering tourism coming from China, including a visit by 12’000 Chinese travelers – an event extensively covered within Switzerland due to its exceptional number – up to recent times in which non-European tourists are almost absent from the country. The research aims at identifying the main themes being voiced in newspaper articles. It also tackles the themes mentioned in user-generated comments on Facebook on the same articles.

## Introduction

Chinese Outbound Tourism (COT) is an important tourism source market for many destinations globally [1]. For Switzerland, COT has great potential because of its high growth rates, the absolute number of guests arriving and their daily spending [2, 3]. In fact, between the years 2005 and 2016, the flow of tourists from China to Switzerland grew by about 560% with regards to the number of overnight stays and about 700% with regards to the number of arrivals [2]. Moreover, studies have identified a notable willingness amongst Chinese citizens to visit Switzerland and a generally positive perception of the country, because of its natural beauty and offer in prestigious luxury goods [4, 5]. By now, Switzerland has hosted guests coming from mainland China for many years, with some noteworthy events taking place, such as the incentive trip of a group of 12’000 Chinese employees, offered by the American direct selling company, Jeunesse Global [6], in May 2019. This resulted in three groups of 4’000 members travelling around Switzerland visiting different destinations one after the other. Notably, one destination they visited was Lucerne, which was extensively covered in Swiss media. According to Switzerland Tourism [7] the trip generated approximately “170 media reports, with over 64 million media contacts”. However, if in the past a major upcoming trend within tourism had been the emergence and growth of the COT market, Covid-19 and the lack of international tourists at destinations [8], has potentially impacted narratives and discussions around the topic.

**Fig. 1.**
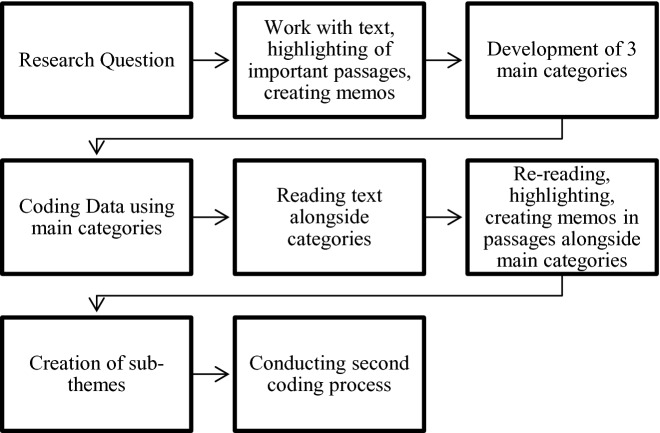
Coding Process based on Kuckartz [32, p.70]

**Fig. 2.**
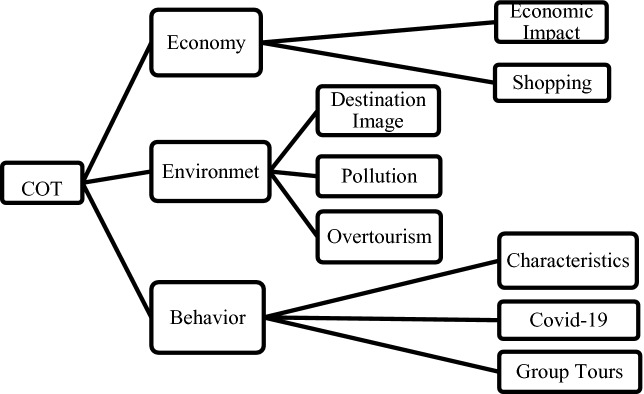
Themes & Sub-themes

**Table 1.** Themes & Sub-themes Article *Neue Zürcher Zeitung*

Theme	Sub-theme	Description	Example (Source: *Neue Zürcher Zeitung*, May 2019)
*Behavior*	*Group tours*	References to group tours, their size, travel itinerary, typical routes, typical behavior or the demographics assigned to group tourists coming from China etc. In contrast to this would be any kind of reference to individual travel behavior by Chinese tourists	“A total of 12,000 employees of the cosmetics company Jeunesse Global were piloted through Switzerland in three waves. Apart from Lucerne, the group visited other hotspots such as the Rhine Falls in Schaffhausen and the Aare Gorge”
*Environment*	*Overtourism*	References to overcrowding, congestions or simply mentioning the word (e.g. in title) etc.	“Many locals feared that this rush would lead to congested streets and traffic jams”

**Table 2.** Themes & Sub-themes Article *Luzerner Zeitung*

Theme	Subtheme	Description	Example (Source: *Luzerner Zeitung*, February 2020)
*Economy*	*Shopping*	References to specific shopping habits/behavior/preferences or facts assigned to Chinese visitors etc.	“We expect a massive cut in the mechanical watches popular in China in particular, says co-owner Robert Casagrande”
	*Economic impact*	References to impacts of COT on local/regional/national economy etc.	“The host shrugs its shoulders. ‘What shall we do? We are currently experiencing a 70 to 80 percent drop in sales,’ the man says”
*Behavior*	*Group tours*	See description above.	“(…) where otherwise tourist busses drive up every minute and women and men from Asia busily get in and out”
	*Covid*-*19*	References to the effects of the Covid-19 outbreak on COT and local destinations etc.	“Lucerne currently presents itself quite deserted. This since the Chinese government stopped group travel to Europe because of the corona virus”
*Environment*	*Destination image*	Reference to the effect of COT on the destination image etc.	“The picture seems almost idyllic. Lucerne currently presents itself quite deserted”
	*Overtourism*	See description above	“. . . untenable conditions by bus traffic. . .”

The study of trends, motivations, attitudes and behaviors of tourists travelling to different destinations is a popular research topic within the field of tourism, and naturally this also accounts for COT [9–12]. However, the following exploratory study aims at adding to the research on COT by considering a different perspective. Moreover, it is part of a bigger project exploring the public discourse around COT to Europe in Western mass media and on social media before and after the COVID-19 outbreak. The findings of this project could serve the sustainable and responsible reestablishment of international tourism coming to Europe. It is important to understand that the research is not about Chinese thought, culture or politics, but rather the view on COT in Western media and on social media. Moreover, the “consolidation of news coverage with user-generated content in social media” [13, p. 14] will add to tourism research concerning the topic of “host-tourist-relationships”.

For a first step, the focus of this exploratory study is mainly on Swiss-German media outlets and the articles published about COT in weekly and daily print newspapers. Next, the user’s opinions that are being expressed about mainland Chinese tourists on the social media platform Facebook in reaction to the images portrayed by the selected media articles are examined. The research question that this study wants to answer is the following*: what kind of themes emerge within the Swiss*-*German mass media coverage on COT and on social media?* For the context of this study, COT involves all the activities that citizens of the People’s Republic of China undertake while traveling to and staying at destinations outside their country of residency for more than one night. Regarding the Swiss mass media landscape, it is notable that, even though the country is small in size with small cantons, it has a high newspaper density if compared to other countries and is furthermore characterized by a strong regional orientation and multilingualism [14, 15].

## Literature Review

### News Media Analysis and Tourism

Media analysis deals with media products like news media and analyzes their constructions of meaning, structures and aesthetic form [16]. It verifies or falsifies the general statements of media theory and media historiography by examining them in relation with a concrete subject [16]. The importance of news media analysis in tourism research has been widely acknowledged in terms of the agenda setting effect of mass media on public opinion formation [17] about tourism, as well as understanding tourism impacts, which influence tourism development and governance [18, 19]. Studies can range from the discussion and analysis of the media’s role in influencing the formation of destination images [20, 21], producing important insights around topics such as “overtourism” by applying methods such as content or thematic narrative analysis [22, 23], to employing frame analysis for the identification of main frames regarding the online news media discourse on the “Golden Week” policy in China [24]. A noteworthy example for the media analysis of COT is the study by Hao et al. [13], who applied news media sentiment analysis, computing the general sentiment regarding mainland Chinese tourists covered in over 70,000 newspaper articles published in Hong Kong from 2003 to 2015, validating the results with typical socioeconomic factors and key socioeconomic events that occurred.

### Analysis of UGC and Tourism

User generated content (UGC) is the “content that is produced by users of… [a] medium rather than by media professionals” [25]. This also includes comments posted publicly on social media. In the last decade, a major stream of research in tourism has focused on the analysis of UGC from a visitor perspective, quite often concentrating on online travel reviews, including works of hospitality research [26–28]. According to Marchiori and Cantoni [29], these UGC represent instances of published opinion (not public opinion overall) about a specific topic. This can then influence how a destination or tourism service provider is perceived or UGC shape travel decisions of others [30]. In their review of 122 scientific articles, with the earliest published in 2001, Lu and Stepchenkova [31] concluded that the research revolves mainly around the topics of (1) “service quality”, (2)”destination image and reputation”, (3) “experiences and behavior”, (4) “the persuasive power of UGC” (e.g. for eWOM), and (5) “tourist mobility patterns”. In terms of methodology, content analysis of textual UGC was employed the most. An example regarding COT and UGC analysis is for instance the work published by Hu et al. [4] investigating the reputation of Switzerland as a tourism destination on the Chinese microblogging platform Sina Weibo, again focusing on a demand perspective in tourism. What seems to be missing from the literature are studies on UGC and tourism from the “local” perspective, however future studies on this seem to have been suggested [13], but not notably published [31].

## Methodology

In order to tackle the above stated research question, for this kind of preliminary study, it was decided to apply the method of thematic analysis [32], defining major themes that occur in the articles about COT. This kind of analysis does not give results on opinions expressed in the text, the structure of arguments, nor does it give any kind of indication on the effect of or intention behind the texts that have been analyzed. These can only be hypothesized on, by for instance, contextualizing the results with the troves on news value theory [33–35].

### Sample Building

To kick off the research, it was decided that the newspaper articles that should be analyzed should come from the weekly and daily newspapers with the largest circulation numbers in Switzerland. The preliminary study presented here is on Swiss German media, which address the biggest linguistic region within the country. The statistics on the circulation numbers were taken from the 2019 WEMF “Swiss press circulation bulletin” which summarizes the data from 01.04.2018 until 31.03.2019 [36]. Of those newspapers only those that are present on at least one of three commonly used social media platforms in Switzerland: Facebook, Instagram and/or Twitter were selected. The presence on these social media platforms is essential for the next research steps of the overall project. Finally, one last requirement of selection was the newspaper’s presence on the research and business information tool, Factiva (www.factiva.com). In the end, eight Swiss-German daily (*20 Minuten*, *Die Südostschweiz*, *Tages*-*Anzeiger*, *bz* – *Berner Zeitung*, *Luzerner Zeitung*, *St. Galler Tagblatt*, *Blick* and *Neue Zürcher Zeitung*) and three Swiss weekly newspapers (*SonntagsZeitung*, *Sonntags*-*Blick*, *NZZ am Sonntag*) were selected (n = 11).

### Corpus Selection & Data Cleaning

The period chosen to select the corpus was from 01.01.2019 to 30.06.2020 (in total 18 months). This ensured that the event of May 2019 of 12.000 visitors from China visiting Switzerland and the outbreak of Covid-19 were covered. In order to find content covering COT, the keywords “China” and “Tourism” (in German language) were used in Factiva. In total, 457 articles were found published by the eleven sources, once duplicates had been removed. Next, the first round of data cleaning followed. Only if COT constituted at least 50% of the concerned article, it would be considered as a dominant topic and the article would be kept within the sample. Articles in which tourism was, for example, only mentioned as an industry suffering because of Covid-19 and China was not mentioned in relation with tourism were excluded. In the end, the database amounted to 60 articles. Interestingly, one newspaper (*Sonntags*-*Blick*) did not seem to have published any article where COT was considered a dominant topic.

For comparative reasons it was decided to also search the number of articles only mentioning the keyword “China” (in German language). One can see that the combination of “China” and “Tourism” takes up a rather small amount of mentions in comparison to articles just mentioning “China”. “China” and “Tourism” are mentioned in 3,4% (n = 457) of the sample only mentioning “China” (n = 13.320). Moreover, after the data cleaning only 13,1% (n = 60) out of the articles mentioning “China” and “Tourism” (n = 457) made it into the final sample.

Furthermore, the frequency of the mentions of the words “China” and “Tourism” over the selected time frame of 18 months (in this case with duplicates, n = 487) was considered. Interestingly, one can observe three peaks where the words “China” and “Tourism” were mostly mentioned: the first in May 2019 (in total 42 mentions), the second in July 2019 (in total 32 mentions) and the last in February 2020 (in total 69 mentions). It is striking that May 2019 was the month when the 12.000 visitors from China visited Switzerland, whereas February 2020 was two months after Covid-19 had been officially announced by the World Health Organization, and when daily cases in the Western Pacific reached their first peak [37].

In a second step, the articles were traced back to the Facebook pages of the concerned media outlets. This could be done by manually researching the articles on the different Facebook pages of the media outlets. Facebook was selected as the first platform to collect UGC because it is a frequently used social media platform in Switzerland. In December 2019, 3,5 million users logged into Facebook in Switzerland [38]. Considering that the number of people living in Switzerland in 2019 was 8,606 million [39], 3,5 million Facebook users would amount to roughly 41% of the population. When checking the Facebook page of the media organizations in the sample (on 05.09.2020), the number of followers ranged from 555,633 (*20 Minuten*) to 8,477 (*NZZ am Sonntag)*. Notably, not all of the word-for-word versions of the printed articles could traced back online, yet, the main topics of the online articles and the print articles were the same. As an example, a post about the article on the case of 12.000 tourists by *20 Minuten* (print) published on May 14, 2019 could not be found online on the Facebook page of the organization. However, around the same time, two posts directing to online articles about the same topic were published.

Facebook posts by news media organizations are published online and publicly accessible for any entity with a Facebook account. Nonetheless, for privacy and legal reasons, usernames or profile descriptions of the “commentators” were not included in the data collection and are hence not part of the analysis. Furthermore, in this study, user comments were never replicated word for word, but their content was paraphrased. For the purpose of this study, only two posts about articles, which were posted in their “original” print form, from the two “highest” peaks of *frequency of mentions* were selected to be presented further. This was done to put emphasis on the qualitative methodological approach of the study and underline its exploratory nature. Furthermore, both articles deal with subjects related to the city of Lucerne, which was also frequently mentioned in the media discourse surrounding the visit of 12’000 Chinese tourists visiting Switzerland in May 2019. Issues such as the discrepancies between the publication of print and online versions of articles (as described above) are still to be solved and currently represent significant issues in terms of the generalization of results.

The first post was about an article that was published on May 24, 2019 by the *Neue Zürcher Zeitung* concerning “the case of 12’000 visitors to Lucerne” published after the event occurred (14.05.2020). It was concurrently published on the newspaper’s Facebook page (254.585 followers by 05.09.2020) on May 24, 2019, and received 71 reactions (likes, dislikes etc.), 73 comments and 12 shares (until 05.09.2020). For the analysis, only the first level of user generated comments was considered, while the comments to the comments were excluded. In total, 32 comments were analyzed.

The second post was about an article that was published on February 8, 2020 by the *Luzerner Zeitung* with a headline indicating that Chinese tourists were not present on one of the city’s main attractions (a square). Concurrently, a post about the article was published on their Facebook page (31.783 followers by 05.09.2020); it received 524 reactions, 223 comments and 51 shares. In total, all 52 first-level comments were analyzed.

### Thematic Analysis

Thematic analysis can be considered a basic method of qualitative text analysis and is used in this context as an exploratory and descriptive approach [32]. As a category-based method the text was analyzed according to different themes, which are presented to the reader [32]. Moreover, according to Kuckartz [32, p.70] thematic analysis is a “method that reduces content”. For this study, after the data was cleaned, the text was thoroughly read. Afterwards an inductive theme building approach followed. The first number of categories derived from this stage is rather rough and manageable. In a next step, the main categories are divided into sub-themes. In this study, this process is shown by the in-depth description of the analysis of the two articles chosen to exemplify the research. Moreover, a theme was coded as major if it was described in at least one third of the article. In order to collect, highlight, code and analyze the different articles, the qualitative data analysis software nVivo was used. Figure 1 (see below) shows the sequential process applied, which was based on Kuckartz [32, p.70].

This process was first applied to the 60 selected news media articles. In a second step, the user-generated comments of two articles on Facebook were coded using the same process. The description of the results is presented hereafter.

## Results

For the 60 articles three umbrella categories emerged: *economy*, *environment* and *behavior*. The text passages analyzed can range from very descriptive to more opinionated sections, depending on the kind of article, newspaper or author. Examples for them can be found below in Table 10.1007/978-3-030-65785-7_1 and 10.1007/978-3-030-65785-7_2. The passages have been translated from German into English by the author. Moreover, a passage can also be assigned to two or more themes.

*Economy* references are about the impact or effect of COT on local, regional,

national or international economy. This can include the enumeration of economic figures, discussions around spending capacity, description of shopping behavior etc.

*Environment* references concern the impact and effect of COT on regional, national and international environment. They can include the discussion on specific ecological issues but also the wearing out or over usage of urban and rural infrastructure (e.g. topic of overtourism).

*Behavior* references describe one or many specific tourism behaviors supposedly assigned to Chinese tourists. This can include stating facts on group tour behavior or simply the itinerary, the enumeration of facts on group sizes but also the reproduction of specific stereotypes. Here it is important to remember that these descriptions are made by individual journalists and represent their views, not the ones of the authors of this paper.

For this preliminary study it was possible to identify references for all three categories, however the category “environment” seems to be less discussed. It was only possible to identify a small amount of references to this theme and only in two articles it was coded as a major theme.

### Themes Mentioned Before the Covid-19 Outbreak

For the article published before the Covid-19 outbreak, the reporting is dominantly about *behavior* and *environment*. No references to *economy* could be identified. Table 10.1007/978-3-030-65785-7_1 (see below) depicts the sub-themes that arose through further analysis. The article is also concerned with the “overtourism aspects” when it comes to COT to Switzerland.

Regarding the analysis of UGC on Facebook, more sub-themes emerged. Nonetheless, with the material derived from Facebook there were also specific types of comments emerging from the corpus, which need specific mentioning since they were excluded from the analysis. These were (1) “tags” linking other users to the post. Presumably, this is done by one user to make another user pay attention to the content of the post; (2) “Incomprehensible comments”, meaning that the linguistic content of the comment could not be identified by the authors (e.g. unclear spelling, unknown language); (3) “Links” are comments that are not about COT per se and therefore not part of the research question. An example is a comment remarking that tourism in Switzerland is better than “asylum abuse”; (4) “Author Comments”, which are comments by the organization that published the post about the article (e.g. giving a correction, providing further details, tagging another article related to similar topics mentioned in the article). (5) “Other”, which refers to comments that require a specific cultural repertoire to understand its meaning, being of exceedingly high interpretative nature, which might harm the reliability of coding. An example of this is a user quoting Goethe’s 1797 “The Sorcere’s Apprentice”, not giving any more context (Source: Facebook, @nzz 2019).

In general, regarding the most dominant theme within the UGC, the comments relate to the environmental impact of COT, mostly concerned around the subject of *pollution* and *overtourism*. *Pollution* is a sub-theme that includes all references of COT and its impact on environmental pollution (e.g. air, land etc.). References can be made to CO2 emissions caused by flying to Europe or using buses as transportation means. The environmental comments are followed by comments regarding tourism *behavior* where one new sub-theme called *characteristics* emerged. The sub-theme concerns specific mental, moral and cultural qualities users assign to Chinese tourists. An example of this would be the comment of a user stating that the residents prefer intelligent Chinese guests over guests coming from Arab countries. In this case, the user seems to assign Chinese visitors the attribute that they are intelligent, in comparison to other visitor segments. The implicit meaning of these kinds of statements will be further analyzed when discourse analysis will be applied. Comments regarding the economic effect of COT are not absent from the corpus of UGC but appear less frequently than comments on the other to sub-themes.

### Themes Mentioned Since the Covid-19 Outbreak

For the article published after the Covid-19 outbreak there is a dominant reporting on the economic impact of COT on the tourism industry (or better how the lack of COT will impact the Swiss tourism industry). Moreover, there are references to the tourism *behavior* of Chinese visitors, mostly referring to “typical group tour behavior” that existed before the Covid-19 outbreak. References on *environment* are less poignant. Table 10.1007/978-3-030-65785-7_2 (see below) shows the sub-themes that have emerged for the specific article.

All three main categories that emerge when speaking about COT could again be identified in the user generated comments on Facebook. Users are again discussing the environmental impact of COT to a destination like Lucerne, mostly commenting regarding *pollution* and *overtourism*. References to *overtourism* often speak of the lack there of. An example for a comment regarding *pollution* states that a terrible virus like Covid-19 can have positive effects and that the climate benefits from it, thanks to the limited mobility of millions. The environmental comments are discussed most dominantly, which are then followed by topics relating to economic impacts of COT, specifically, when it comes to the impact of COT on the national tourism industry in comparison to the local industry. The comments posted underneath this article relating to tourism behavior of COT are less discussed in absolute terms. This presents a difference to comments published before the COVID-19 outbreak. Moreover, there seem to be more comments linking to other topics that are not related to COT to Switzerland, for example, commenting on the nature of the Swiss tourism industry in general.

## Conclusions

The results of this preliminary study show that there are three major themes that can be identified within the public discourse around COT in “old” and “new” media. Moreover, sub-themes of these three major topics can be found in articles and UGC on social media. Interestingly, so far there seem to be differences of the dominance of themes and sub-themes addressed in the mass media and addressed in the comments reacting to the articles on social media. All in all, Fig. 2 (see below) shows all the themes and sub-themes that have emerged from the analysis of the two articles and their UGC, no matter the time frame (before or during Covid-19).

In the past years a major emerging topic within tourism has been so-called “overtourism”. The results of this preliminary study show that the issue is not only a topic for researchers and policy makers but has become a hot issue within the public debate and that Covid-19 has dramatically impacted such discussions. The question that also arises from this research is: how are we planning on dealing with so-called “undertourism”?

Due to the preliminary nature of the empirical research, it has clear limitations regarding selected time frame, sample size, scaling and possibility of generalizing its results. Moreover, given that daily and weekly press has different editorial proposals, the authors are considering in future studies to separate these media outlets due to the nature of their publication frequency. The reason why they were considered as part of a single material in this study was due to the selection of newspapers based on their distribution numbers and not publication frequency. Additionally, quantitative methods will potentially also be integrated within this research (e.g. text mining using R) to enable an analysis of much larger corpora.

Nonetheless, the exploratory identification of major themes is crucial for the broader context of the research project related to the analysis of the public discourse around COT to Switzerland, because, in the end, these collected blocks of thematic references will serve as the basis for the critical analysis of different sentiments, narratives and arguments presented in the public discourse about COT to Europe. Of course, due to the current pandemic, it is unclear when and to what extend COT will return. Nonetheless, the authors believe that this kind of research will contribute to the “responsible and safe restart of tourism” [40], pushing for new findings in the field of “tourism-host-relationship” research, which will not only benefit academia but should present important results for any kind of political or private stakeholders that are concerned with the sustainable development of the tourism industry in Europe.

All kinds of stakeholders in the tourism sector (including DMO’s) could greatly benefit from the results of this kind of research because even though they will not show the opinions of their locals, they will show which kind of narratives (including racist stereotypes), might currently polarize parts of the public agenda and, that could influence public opinion on tourism and COT. The knowledge gained from this research project should not only help to put in place the right kind of communication strategy to tourists but maybe even more importantly to the locals, minimizing stereotyped views, especially after Covid-19.
